# Loss of DNA mismatch repair facilitates reactivation of a reporter plasmid damaged by cisplatin

**DOI:** 10.1038/sj.bjc.6690412

**Published:** 1999-05

**Authors:** B Cenni, H-K Kim, G J Bubley, S Aebi, D Fink, B A Teicher, S B Howell, R D Christen

**Affiliations:** Department of Medicine 0058, University of California San Diego, La Jolla, CA 92093-0058, USA; Beth Israel Deaconess Hospital, Harvard Medical School, Boston, MA, 02115, USA; Division of Cancer Pharmacology, Dana Farber Cancer Institute, Boston, MA, 02115, USA

**Keywords:** cisplatin, DNA mismatch repair, hMLH1, hMSH2, colon cancer, endometrial cancer

## Abstract

In addition to recognizing and repairing mismatched bases in DNA, the mismatch repair (MMR) system also detects cisplatin DNA adducts and loss of MMR results in resistance to cisplatin. A comparison was made of the ability of MMR-proficient and -deficient cells to remove cisplatin adducts from their genome and to reactivate a transiently transfected plasmid that had previously been inactivated by cisplatin to express the firefly luciferase enzyme. MMR deficiency due to loss of hMLH1 function did not change the extent of platinum (Pt) accumulation or kinetics of removal from total cellular DNA. However, MMR-deficient cells, lacking either hMLH1 or hMSH2, generated twofold more luciferase activity from a cisplatin-damaged reporter plasmid than their MMR-proficient counterparts. Thus, detection of the cisplatin adducts by the MMR system reduced the efficiency of reactivation of the damaged luciferase gene compared to cells lacking this detector. The twofold reduction in reactivation efficiency was of the same order of magnitude as the difference in cisplatin sensitivity between the MMR-proficient and -deficient cells. We conclude that although MMR-proficient and -deficient cells remove Pt from their genome at equal rates, the loss of a functional MMR system facilitates the reactivation of a cisplatin-damaged reporter gene. © 1999 Cancer Research Campaign


*British Journal of Cancer* (1999) **80**(5/6), 699–704
© 1999 Cancer Research Campaign
Article no. bjoc.1998.0412

### Loss of DNA mismatch repair facilitates reactivation of a reporter plasmid damaged by cisplatin

**B Cenni[1,†], H-K Kim[1], GJ Bubley[2], S Aebi[1], D Fink[1], BA Teicher[3,*], SB Howell[1] and RD Christen[1]**

[1]Department of Medicine 0058, University of California San Diego, 9500 Gilman Drive, La Jolla, CA 92093-0058, USA; [2]Beth Israel Deaconess Hospital, Harvard Medical School, Boston, MA 02115, USA; [3]Division of Cancer Pharmacology, Dana Farber Cancer Institute, Harvard Medical School, Boston, MA 02115, USA

**Summary** In addition to recognizing and repairing mismatched bases in DNA, the mismatch repair (MMR) system also detects cisplatin DNA adducts and loss of MMR results in resistance to cisplatin. A comparison was made of the ability of MMR-proficient and -deficient cells to remove cisplatin adducts from their genome and to reactivate a transiently transfected plasmid that had previously been inactivated by cisplatin to express the firefly luciferase enzyme. MMR deficiency due to loss of hMLH1 function did not change the extent of platinum (Pt) accumulation or kinetics of removal from total cellular DNA. However, MMR-deficient cells, lacking either hMLH1 or hMSH2, generated twofold more luciferase activity from a cisplatin-damaged reporter plasmid than their MMR-proficient counterparts. Thus, detection of the cisplatin adducts by the MMR system reduced the efficiency of reactivation of the damaged luciferase gene compared to cells lacking this detector. The twofold reduction in reactivation efficiency was of the same order of magnitude as the difference in cisplatin sensitivity between the MMR-proficient and -deficient cells. We conclude that although MMR-proficient and -deficient cells remove Pt from their genome at equal rates, the loss of a functional MMR system facilitates the reactivation of a cisplatin-damaged reporter gene.

**Keywords:** cisplatin; DNA mismatch repair; hMLH1; hMSH2; colon cancer; endometrial cancer

Cisplatin is a widely used chemotherapeutic drug that has served as the basis for development of subsequent generations of platinum-coordination compounds. Its mechanism of cytotoxicity is the formation of a variety of DNA adducts of which the covalent 1,2 intrastrand cross-link between two adjacent guanines is the most abundant (reviewed in Zamble and Lippard, 1995). Acquired resistance to cisplatin occurs frequently during treatment and is important due to the narrow therapeutic index of this drug. Small changes in sensitivity, in the range of twofold, are sufficient to account for the failure of treatment (Andrews et al, 1990; Fink et al, 1997).

The proteins involved in DNA mismatch repair (MMR) are evolutionarily conserved. The MMR system detects and repairs frameshifts, replication errors, mainly base mismatches, and regulates recombination events (Kolodner, 1995). Interestingly, the MMR system is also involved in the detection of DNA damage produced by 6-thioguanine and methylating agents, as well as cisplatin and carboplatin (Kat et al, 1993; Hawn et al, 1995; Aebi et al, 1996; Drummond et al, 1996; Fink et al, 1996). It has been known for some time that loss of MMR results in high level resistance to 6-thioguanine and moderate resistance to a variety of methylating agents, including *N*-methyl-*N′*-nitro-*N*-nitrosoguanidine (MNNG). Recently, we and others have shown that loss of MMR also results in low-level resistance to cisplatin and carboplatin (Aebi et al, 1996; Drummond et al, 1996; Fink et al, 1996). In the case of cisplatin, it has previously been shown that hMSH2 is a component of the protein complex that binds to DNA-containing cisplatin adducts (Duckett et al, 1996; Fink et al, 1996; Mello et al, 1996), and it has been suggested that the MMR proteins serve as a detector system for the presence of DNA damage (Hawn et al, 1995; Kat et al, 1993). The repair of mismatched bases by the MMR system involves incision of the mismatch-containing strand, either upstream or downstream of the mismatch, excinuclease-helicase-mediated removal of a portion of the incised strand creating a gap, and then filling of the gap and religation by DNA polymerase and ligase (reviewed in Kolodner, 1995). Many of these steps are similar to those performed by the nucleotide excision repair system, a DNA repair system that is known to remove cisplatin adducts from DNA (Zamble and Lippard, 1995).

We sought to determine whether the MMR system is involved in the removal of cisplatin adducts from DNA by comparing the ability of MMR-proficient and -deficient cells of the same genetic background to form and remove adducts in endogenous DNA and to reactivate expression of the luciferase gene from a transiently transfected cisplatin-damaged plasmid. We report here that loss of MMR had no effect on the extent of cisplatin adduct formation or the kinetics of adduct removal from genomic DNA as measured by atomic absorption spectroscopy, but that, contrary to expectation, loss of MMR facilitated the expression of a reporter gene disabled by treatment with cisplatin.

### MATERIALS AND METHODS

### Cell lines and chemicals

The cell lines HCT116+ch2 (clone HCT116/2–1) and HCT116+ch3 (clone HCT116/3–6), derived from the hMLH1-deficient human colorectal adenocarcinoma cell line HCT116 by

*Received 15 May 1998*
*Revised 10 November 1998*
*Accepted 17 November 1998*

*Correspondence to:* RD Christen

Bruno Cenni and Heung-Ki Kim contributed equally to this work.

Present addresses: *Lilly Research Laboratories, Lilly Corporate Center, DC 0540, Indianapolis, IN 46285, USA; †University of Geneva, Department of Cell Biology, Sciences III, 1211 Geneva 4, Switzerland

**Table 1** Platinum content of genomic DNA as a function of time after cisplatin exposure

| | 40 μM cisplatin | | | 80 μM cisplatin | | |
|---|---|---|---|---|---|---|
| Time (h) | HCT116 | +ch2 | +ch3 | HCT116 | +ch2 | +ch3 |
| 0 | 100 | 100 | 100 | 100 | 100 | 100 |
| 6 | 12.0 ± 3.5 | 12.1 ± 3.6 | 10.5 ± 2.7 | 13.1 ± 2.7 | 12.2 ± 2.1 | 11.3 ± 3.3 |
| 20 | 6.4 ± 2.8 | 4.3 ± 1.7 | 5.3 ± 1.8 | 7.2 ± 3.0 | 6.8 ± 2.2 | 6.6 ± 2.0 |
| 28 | 1.1 ± 0.3 | 1.2 ± 0.2 | 0.9 ± 0.3 | 1.6 ± 0.5 | 1.8 ± 0.7 | 1.7 ± 1.3 |

The rates of platinum removal were determined in HCT116 sublines at 0, 6, 20 and 28 h after the end of 1 h exposure to 40 and 80 μM cisplatin. Initial adduct levels were the same in all HCT116 sublines, i.e. 384 fmol $\mu g^{-1}$ DNA and 650 fmol $\mu g^{-1}$ DNA following exposure to 40 μM and 80 μM cisplatin respectively. Values represent mean ± s.d. (*n* = 3) per cent of the initial content at the end of the 1 h treatment with cisplatin. There was no significant difference between MMR-proficient and -deficient cells in the rate of platinum removal over time.

complementation with chromosomes 2 and 3, respectively, were obtained from Drs CR Boland, M Koi and TA Kunkel. Complementation with chromosome 3 provides a wild-type copy of hMLH1 that renders the HCT116+ch3 cells MMR-proficient (Koi et al, 1994). The hMSH2-deficient human endometrial carcinoma cell line HEC59 and its subline HEC59+ch2 (clone HEC59/2–4), complemented with chromosome 2, were also provided by Drs CR Boland, M Koi and TA Kunkel. In the HEC59+ch2 cells, the chromosome 2 complementation restores wild-type hMSH2 and MMR function (Umar et al, 1997). The cells were grown as previously described (Aebi et al, 1996). The status of expression of hMLH1 and hMSH2 was confirmed by Western blot. Cisplatin was obtained from Sigma (St Louis, MO, USA) and dissolved in 0.9% (w/v) saline. Lipofectin was purchased from Life Technologies (Gaithersburg, MD, USA).

### Cellular pharmacology

The effect of MMR on the repair of cisplatin-damaged DNA was compared using two pairs of cell lines. The HCT116-derived sublines differed with respect to MMR activity due to the loss of hMLH1 function, and the HEC59 cells due to the loss of hMSH2 function. The HCT116 cells contain a hemizygous mutation in hMLH1 resulting in a truncated, non-functional protein (Boyer et al, 1995; Carethers et al, 1996). Thus far, complementation of hMLH1 and hMSH2 defects by expression of these genes from a vector has not been reported by any laboratory; however, successful complementation has been achieved using whole chromosomes. The HCT116+ch3 subline is MMR-proficient due to complementation with a wild-type copy of hMLH1 on chromosome 3; the HCT116+ch2 subline is complemented with chromosome 2 and is MMR-deficient (Koi et al, 1994; Carethers et al, 1996). Similarly, the HEC59 cells are mutated at different loci on both alleles of hMSH2 and are deficient in MMR activity (Boyer et al, 1995); the HEC59+ch2 subline complemented with a wild-type copy of hMSH2 on chromosome 2 is MMR-proficient (Umar et al, 1997). The MMR-deficient HCT116 cells are 2.1-fold resistant to cisplatin when compared to the MMR-proficient HCT116+ch3 cells in clonogenic assays, and the MMR-deficient HEC59 cells are 1.8-fold more resistant to cisplatin than the MMR-proficient HEC59+ch2 cells (Fink et al, 1996). The complemented cells grown in G418 have remained stable for more than 2 years in culture, and repeat clonogenic assays confirmed these differences in cisplatin sensitivity (data not shown).

### Assay of platinum adducts in DNA

The extent of DNA platination was measured by exposing exponentially growing cells for 1 h to 100 μM cisplatin; the cells were then washed with cold phosphate-buffered saline (PBS) and lysed in a buffer containing 1% sodium dodecyl sulphate (SDS), 2.6 M sodium chloride, 0.3 M EDTA pH 8.0. DNA was isolated by phenol–chloroform extraction and dissolved in buffer containing 10 mM Tris and 1 mM EDTA pH 8.0. Aliquots of the DNA were digested in 1 M hydrochloric acid at 75°C for 2 h and the hydrolysate was used for the quantitation of platinum (Pt) by flameless atomic absorption spectrophotometry (Perkin-Elmer Model 2380). The rate of cisplatin adduct removal was measured in cells that were exposed for 1 h to 40 and 80 μM cisplatin and harvested 0, 6, 20 and 28 h after the end of exposure. The Pt content of the DNA was measured by atomic absorption spectroscopy as described above.

### Plasmid reactivation assay

A plasmid carrying a 2.4 kb fragment from pB/LUC that included the 1.6-kb firefly luciferase cDNA was prepared by ligating a SalI/NotI fragment that contained the luciferase coding region into the 6.9-kb mammalian expression vector pKEX-2-XR (Rittner et al, 1991) placing the luciferase expression under control of the cytomegalovirus (CMV) promoter. One to 4 mg of plasmid DNA was dissolved in buffer containing 10 mM Tris and 1 mM EDTA pH 7.4 and incubated with 5 μM cisplatin at 37°C for 3 h. The platinated DNA was then purified by ethanol precipitation and unreacted drug was removed by passage of the DNA through a G50 Sephadex column. This procedure resulted in plasmid DNA that was > 90% supercoiled as verified by gel electrophoresis. The platination procedure yielded 1.5 ± 1.4 pg $\mu g^{-1}$ DNA which is equivalent to 9.3 adducts per plasmid or 3.2 adducts per Luc coding region and promoter. Similar levels of platination have previously been shown not to affect the efficiency of transfection (Eastman and Schulte, 1988).

Equal number of cells (i.e. 200 000 per well) were transfected in serum-free medium with 1 μg platinated or unplatinated pKEX-2-XR-Luc in combination with 5 μl lipofectin for a period of 5 h. Intra-assay variability was minimized by using one lipofectin mixture for all samples in each experiment. Subsequently, the DNA was washed off and fresh medium was added. At various time points after transfection, triplicate samples were washed with ice-cold PBS and then lysed in a solution containing 1% Triton

 *© Cancer Research Campaign 1999*

X-100, 15 mM $MgSO_4$, 4 mM EGTA, 1 mM dithiothreitol, and 25 mM glycylglycine at pH 7.8 for 10 min. After centrifugation for 5 minutes at 16 000 ***g***, aliquots of the cleared lysate were assayed for luciferase activity as previously described (Brasier et al, 1989). The generation of luciferase activity as a function of time was compared for cells transfected with the unplatinated versus platinated vector (Eastman and Schulte, 1988). To control for variation in transfection efficiency between experiments, luciferase activity was expressed as percent of maximum activity attained in each experiment. In each cell line, the area under the curve of luciferase activity versus time was computed up to the time of maximal activity which was 36 and 20 h for HCT116 and HEC59 cells respectively. The efficiency of plasmid reactivation was calculated as the ratio of the area under the curve of the platinated vector to the area under the curve of the unplatinated plasmid.

### RESULTS

### Effect of MMR on platinum adduct formation and removal

We have previously shown that after a 1 h incubation in 100 μM cisplatin the HCT116+ch2 and HCT116+ch3 cells do not differ significantly in their total cellular uptake of Pt with accumulation being 303 ± 58 (s.d.) fmol $\mu g^{-1}$ protein and 289 ± 82 (s.d.) fmol $\mu g^{-1}$ protein in the two cell lines respectively ($P = 0.75$, two-tailed *t*-test, $n = 4$) (Aebi et al, 1997). Thus, resistance to cisplatin in the HCT116+ch2 cells is not due to reduced drug uptake. Likewise, the extent of DNA platination was similar in the two cell lines (Aebi et al, 1997).

In order to determine whether loss of MMR altered the kinetics of adduct removal from the whole genome, the Pt removal rates were measured in the HCT116 cell lines at 0, 6, 20 and 28 h after the end of a 1 h exposure to 40 and 80 μM cisplatin. As shown in Table 1, HCT116 cells and their chromosome-complemented sublines demonstrated a rapid decrease in adduct content over the first 6 h following exposure to both cisplatin concentrations, and the kinetics were similar to those previously reported for cisplatin adduct removal (Dijt et al, 1988; Eastman and Schulte, 1988; Jones et al, 1991). However, there was no significant difference between MMR-proficient and -deficient cells in the rate of platinum removal over time.

### Effect of MMR on plasmid reactivation

The effect of loss of MMR on the function of a gene inactivated by cisplatin adducts was examined by comparing the ability of MMR-proficient and -deficient cells to express luciferase from a platinated plasmid-transfected into the cell. Figure 1 shows that luciferase activity appeared in both the MMR-proficient and -deficient HCT116 sublines with the same kinetics when they were transfected with non-platinated vector. Maximum luciferase activity was reached at 36 and 20 h in HCT116 and HEC59 cells, respectively. When the platinated vector was transfected into the MMR-deficient HCT116+ch2 subline, there was little impairment in the generation of luciferase activity (Figure 1A). However, when the same platinated vector was transfected into the MMR-proficient HCT116+ch3 subline, both the rate of appearance of the luciferase activity and the maximal activity attained over the whole observation period were reduced (Figure 1B). A similar pattern was observed in the HEC59 system (Figure 2). The kinetics of appearance of luciferase activity was the same in the HEC59 and HEC59+ch2 cells in the absence of vector platination. However, the MMR-proficient HEC59+ch2 cells were less capable of generating luciferase activity from the platinated vector than the MMR-deficient HEC59 cells.

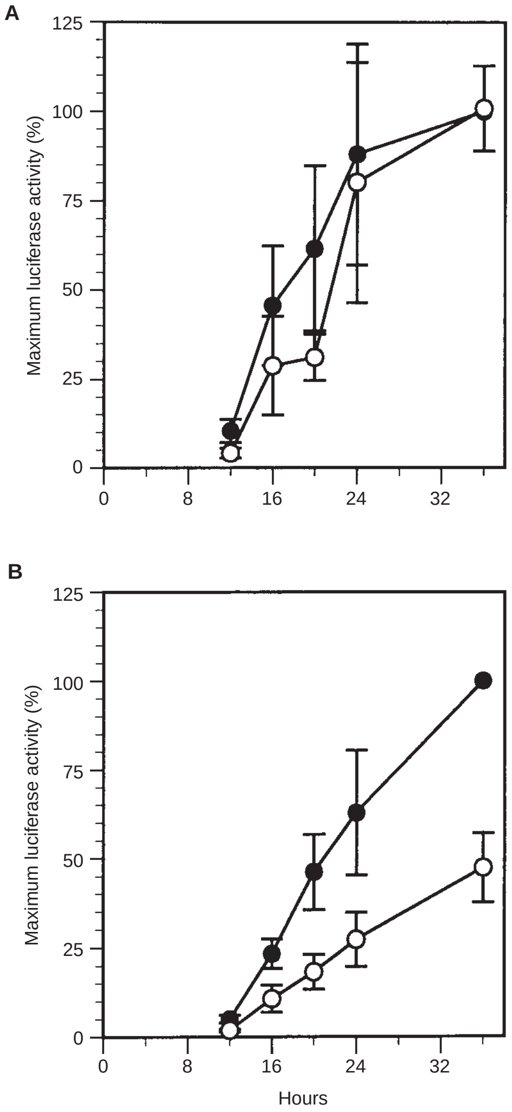

**Figure 1** Luciferase activity as a function of time in HCT116 cells. Luciferase activity was determined following transfection of pKEX-2-XR-Luc in MMR-deficient HCT116+ch2 cells (**A**) and MMR-proficient HCT116+ch3 cells (**B**). (●), non-platinated vector; (○), platinated vector. Luciferase activity is expressed as per cent of maximum luciferase activity generated by the unplatinated vector at 36 h. Data points represent the mean ± s.e.m. of three experiments each performed with triplicate transfections for every time point

Figure 3 shows that the efficiency of reactivation, calculated from all three sets of experiments as the ratio of the area under the curve of luciferase activity versus time for the platinated plasmid divided by that for the unplatinated plasmid in each cell line, was consistently lower in the MMR-proficient cells than in their MMR-deficient counterparts in both cell systems. The MMR-proficient HCT116+ch3 cells were 2.1 ± 0.7-fold (± s.d., $n = 3$) less efficient at expressing luciferase from the platinated vector than their MMR-deficient HCT116+ch2 counterparts ($P = 0.0355$ by paired *t*-test for the comparison of MMR-proficient vs

© Cancer Research Campaign 1999

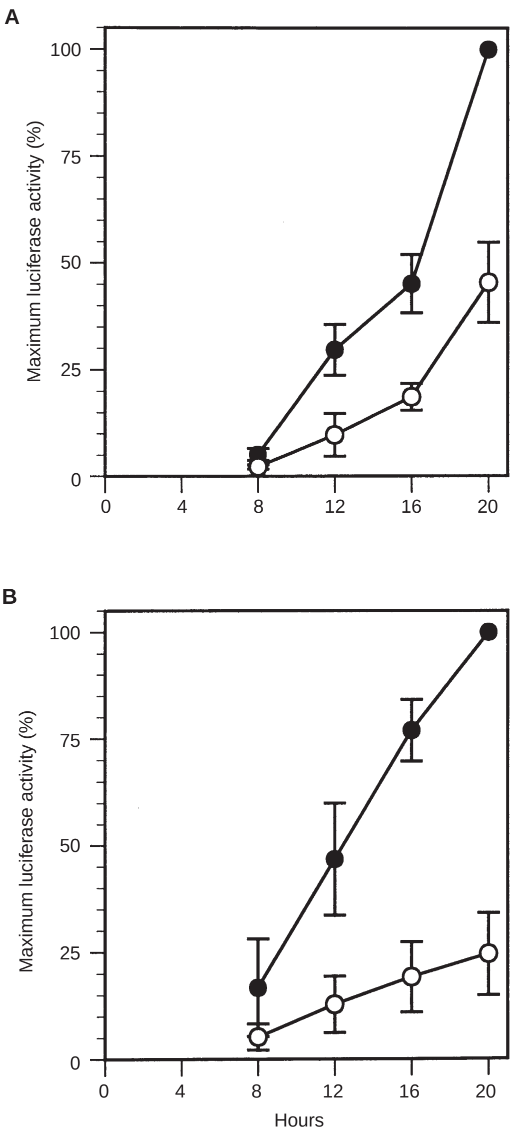

**Figure 2** Luciferase activity as a function of time in HEC59 cells. Luciferase activity was determined following transfection of pKEX-2-XR-Luc in MMR-deficient HEC59 cells (**A**) and MMR-proficient HEC59+ch2 cells (**B**). (●), non-platinated vector; (○), platinated vector. Luciferase activity is expressed as percent of maximum luciferase activity generated by the unplatinated vector at 20 h. Data points represent the mean ± s.e.m. of three experiments each performed with triplicate transfections for every time point

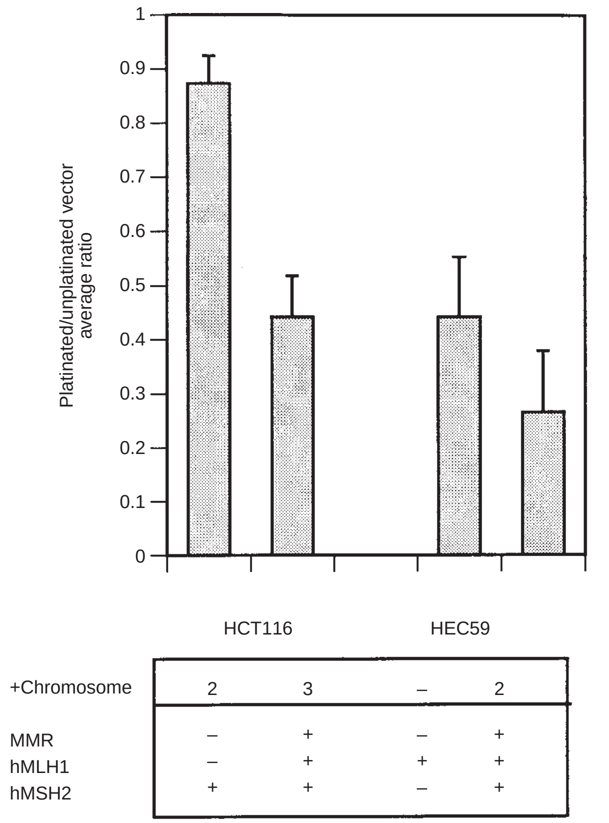

**Figure 3** Efficiency of the generation of luciferase activity in MMR-deficient and -proficient cells. The efficiency of plasmid reactivation is expressed as the ratio of the area under the curve of luciferase activity over time for the platinated vector divided by the area under the curve for the unplatinated vector in the same cells. Bars indicate mean ± s.e.m. ($n = 3$). MMR-proficient HCT116+ch3 cells were less efficient at expressing luciferase from the platinated vector compared to MMR-deficient HCT116+ch2 cells ($P = 0.0355$ by paired *t*-test). Similarly, MMR-proficient HEC59+ch3 cells were less efficient at expressing luciferase compared to MMR-deficient HEC59 cells ($P = 0.002$ by paired *t*-test)

-deficient HCT116 cells). In the HEC59 system, the MMR-proficient HEC59+ch2 cells were 1.9 ± 0.6-fold (± s.d, $n = 3$) less efficient at expressing luciferase activity compared to MMR-deficient HEC59 cells ($P = 0.002$ by paired *t*-test for the comparison of MMR-proficient vs-deficient HEC59 cells).

### DISCUSSION

The mechanism by which loss of MMR causes resistance to cisplatin is unknown. A current hypothesis is that MMR proteins serve as a detector for DNA damage caused by cisplatin, as they do for damage produced by methylating agents or the incorporation of 6-thioguanine, and that MMR proteins are involved in the generation of a pro-apoptotic signal since loss of MMR in cancer cells results in increased resistance to cisplatin (Branch et al, 1993; Kat et al, 1993; Aebi et al, 1996; Drummond et al, 1996; Fink et al, 1996). It is, however, not known whether simple assembly of part or all of the MMR protein complex on the platinated DNA is sufficient to generate such a signal or whether the apoptosis is activated by additional damage done to the DNA resulting from attempts made by the MMR system to remove the cisplatin adduct. A futile cycle of excision and resynthesis has been suggested as the basis for the cytotoxicity of agents such as MNNG and 6-thioguanine that produce damage recognized by the MMR system (Karran and Bignami, 1994).

Impaired cellular accumulation of cisplatin is a common mechanism of resistance in the majority of cell lines selected for resistance to this drug (Gately and Howell, 1993). However, MMR-deficient HCT116+ch2 and -proficient HCT116+ch3 cells accumulated the same amount of Pt and had the same extent of DNA platination after a 1 h exposure to cisplatin. The fact that the nucleotide excision repair system proteins can both recognize and remove cisplatin adducts begs the question of whether the MMR system is similarly able to remove cisplatin adducts as well as to detect them. The observation that the kinetics of cisplatin adduct removal appeared to be equivalent in the MMR-proficient and

*© Cancer Research Campaign 1999*

-deficient HCT116 cells suggests that this is not the case. Thus, the difference in sensitivity to cisplatin cannot be explained by differential drug uptake or differential cytosolic detoxification of cisplatin prior to its reaction with the DNA, and the mechanism of resistance does not alter the rate of adduct removal from the total genome. One cannot conclude, however, that the MMR system plays no role in the actual removal of cisplatin adducts from the DNA since it has been established that cisplatin adducts are preferentially removed from transcribed genes as compared to the total genome, and that the coding strand is repaired preferentially compared to the non-coding strand (Jones et al, 1991; May et al, 1993). Thus, measurement of total genomic platination may miss important functional differences in the ability of MMR-deficient and -proficient cells to successfully express genes damaged by platination, since assays of total genomic platination do not measure the final completion of the repair process.

The reporter gene reactivation assay has several advantages over total genome Pt measurement as an assay of overall repair. First, generation of luciferase activity reflects repair activity directed to a transcribed gene. Second, the assay measures the ability of the repair systems to complete all steps in the process and actually generate a functional protein. Third, the reporter gene reactivation assay has previously been validated for cisplatin adduct repair (Sheibani et al, 1989; Jennerwein et al, 1991; Parker et al, 1991; Ali-Osman et al, 1994). One limitation of this assay system is that it reflects repair processes occurring in an extrachromosomal segment of DNA rather than in an endogenous gene.

The finding that MMR proficiency resulted in impaired expression of luciferase from the platinated vector was unexpected. The fact that the same result was obtained in two independent cell types, each rendered MMR-deficient by the loss of a different MMR protein, lends credence to the observation. Several explanations are possible. First, successful binding of the MMR complex of proteins to the cisplatin adduct may sterically hinder the ability of nucleotide excision repair proteins to access and process the lesion, as has previously been suggested (Mello et al, 1996). The ability of the nucleotide excision repair system to remove cisplatin adducts has been well-documented, as has the fact that adduct removal by this system is a major determinant of cellular sensitivity to cisplatin (reviewed in Zamble and Lippard, 1995). Thus, steric hindrance by the MMR proteins would be expected to slow repair by the nucleotide excision repair system and reduce generation of luciferase activity. Such a mechanism has been proposed to explain the ability of another group of cisplatin adduct-binding proteins, the HMG proteins, to interfere with adduct repair (Huang et al, 1994). However, in a recent study, (Mu et al, 1997) reported that addition of the hMSH2/hMSH6 heterodimer to a cell-free excision repair system did not impair the ability of the nucleotide excision repair system to remove cisplatin adducts from DNA. The assay system utilized by these investigators measured only the excision nuclease activity in the absence of transcription, and the possibility of a negative interaction between the MMR and nucleotide excision repair systems in assays including transcription needs further investigation.

A second possibility is that, following recognition, the MMR system processes the adduct in some way that impairs transcription, perhaps by damaging the template strand as has been suggested for the 6-thioguanine and MNNG adducts (Karran and Bignami, 1994). The MMR system may incise the strand opposite the adduct resulting, through the action of an exonuclease, in the creation of a gap whose filling is blocked by the persistence of the adduct. Under circumstances where the gapped strand is the template strand this would be expected to diminish transcription.

Finally, a third possible explanation is that the MMR proteins normally prevent RNA polymerase II from bypassing the cisplatin adduct, and that when the MMR system is disabled there is a higher probability of successful bypass transcription. Transcriptional bypass of Pt adducts by RNA polymerase II in a similar reporter plasmid has previously been described, albeit at low levels for cisplatin (Mello et al, 1995). Interestingly, the cisplatin-resistant human ovarian carcinoma cells A2780/CP70 have increased DNA replication bypass of cisplatin adducts compared to the parental A2780 cells (Vaisman et al, 1997), and they have previously been reported to lack hMLH1 expression and MMR function (Drummond et al, 1996). Additionally, the A2780/CP70 cells exhibit increased ability to reactivate a reporter gene (Parker et al, 1991). Further, defects in hMSH6 are associated with increased resistance and enhanced replicative bypass of cisplatin (Vaisman et al, 1998). These findings suggest that the hMutS$\alpha$ heterodimer consisting of hMSH2 and hMSH6 participates in the recognition of cisplatin adducts and that the loss of hMutS$\alpha$ results in resistance to cisplatin by allowing enhanced replicative bypass of cisplatin adducts. Although transcriptional bypass is likely to generate mutant transcripts, a significant fraction of these may carry silent mutations that still permit the synthesis of functional proteins. Thus, successful transcription of damaged genes could explain the reduced toxicity of cisplatin adducts in cells lacking MMR.

Independent of the mechanism, it is of interest that the loss of MMR activity has an effect of similar magnitude on both the efficiency of luciferase expression and the level of cellular resistance to cisplatin. This is consistent with the hypothesis that the enhanced reactivation ability observed in the MMR-deficient cells is mechanistically linked to determinants of cellular resistance.

### ACKNOWLEDGEMENTS

We thank Dr Dennis Gately for the preparation of the pKEX-2-XR-Luc vector and Drs Boland, Koi and Kunkel for generous contribution of the cell lines. This work was supported by fellowship awards to BC from the Swiss National Science Foundation, the Cancer League of Basel, the Ciba-Geigy-Jubiläumsstiftung and the Schweizerische Stiftung für biologisch-medizinische Stipendien, grant 4154 from the Council for Tobacco Research and grants from CAPCURE and the Colleen Gilbert Foundation. KHK was supported by the Saint Paul Hospital of the Catholic University in Seoul, Korea. This work was conducted in part by the Clayton Foundation for Cancer Research–California Division. SBH and RDC are Clayton Foundation investigators.

### REFERENCES

Aebi S, Kurdi-Haidar B, Gordon R, Cenni B, Zheng H, Fink D, Christen RD, Boland CR, Koi M, Fishel R and Howell SB (1996) Loss of DNA mismatch repair in acquired resistance to cisplatin. *Cancer Res* **56**: 3087–3090

Aebi S, Fink D, Gordon R, Kim HK, Zhen H, Fink JL and Howell SB (1997) Resistance to cytotoxic drugs in DNA mismatch repair-deficient cells. *Clin Cancer Res* **3**: 1763–1767

Ali-Osman F, Berger MS, Rairkar A and Stein DE (1994) Enhanced repair of a cisplatin-damaged reporter chloramphenicol-O-acetyltransferase gene and altered activities of DNA polymerases alpha and beta, and DNA ligase in cells of a human malignant glioma following in vivo cisplatin therapy. *J Cell Biochem* **54**: 11–19

© Cancer Research Campaign 1999 

Andrews PA, Jones JA, Varki NM and Howell SB (1990) Rapid emergence of acquired cis-diamminedichloroplatinum(II) resistance in an in vivo model of human ovarian carcinoma. *Cancer Commun* **2**: 93–100

Boyer JC, Umar A, Risinger JI, Lipford JR, Kane M, Yin S, Barrett JC, Kolodner RD and Kunkel TA (1995) Microsatellite instability, mismatch repair deficiency, and genetic defects in human cancer cell lines. *Cancer Res* **55**: 6063–6070

Branch P, Aquilina G, Bignami M and Karran P (1993) Defective mismatch binding and a mutator phenotype in cells tolerant to DNA damage. *Nature* **362**: 652–654

Brasier AR, Tate JE and Habener JF (1989) Optimized use of the firefly luciferase assay as a reporter gene in mammalian cell lines. *Biotechniques* **7**: 1116–1122

Carethers JM, Hawn MT, Chauhan DP, Luce MC, Marra G, Koi M and Boland CR (1996) Competency in mismatch repair prohibits clonal expansion of cancer cells treated with *N*-methyl-*N*′-nitro-*N*-nitrosoguanidine. *J Clin Invest* **98**: 199–206

Dijt FJ, Fichtinger-Schepman AM, Berends F and Reedijk J (1988) Formation and repair of cisplatin-induced adducts to DNA in cultured normal and repair-deficient human fibroblasts. *Cancer Res* **48**: 6058–6062

Drummond JT, Anthoney A, Brown R and Modrich P (1996) Cisplatin and adriamycin resistance are associated with MutLalpha and mismatch repair deficiency in an ovarian tumor cell line. *J Biol Chem* **271**: 19645–19648

Duckett DR, Drummond JT, Murchie AI, Reardon JT, Sancar A, Lilley DM and Modrich P (1996) Human MutSalpha recognizes damaged DNA base pairs containing O6-methylguanine, O4-methylthymine, or the cisplatin-d(GpG) adduct. *Proc Natl Acad Sci USA*, **93**: 6443–6447

Eastman A and Schulte N (1988) Enhanced DNA repair as a mechanism of resistance to cis-diamminedichloroplatinum(II). *Biochemistry* **27**: 4730–4734

Fink D, Nebel S, Aebi S, Zheng H, Cenni B, Nehme A, Christen RD and Howell SB (1996) The role of DNA mismatch repair in platinum drug resistance. *Cancer Res* **56**: 4881–4886

Fink D, Zheng H, Nebel S, Norris PS, Aebi S, Lin TP, Nehme A, Christen RD, Haas M, MacLeod CL and Howell SB (1997) In vitro and in vivo resistance to cisplatin in cells that have lost DNA mismatch repair. *Cancer Res* **57**: 1841–1845

Gately DP and Howell SB (1993) Cellular accumulation of the anticancer agent cisplatin: a review. *Br J Cancer* **67**: 1171–1176

Hawn MT, Umar A, Carethers JM, Marra G, Kunkel TA, Boland CR and Koi M (1995) Evidence for a connection between the mismatch repair system and the G2 cell cycle checkpoint. *Cancer Res* **55**: 3721–3725

Huang JC, Zamble DB, Reardon JT, Lippard SJ and Sancar A (1994) HMG-domain proteins specifically inhibit the repair of the major DNA adduct of the anticancer drug cisplatin by human excision nuclease. *Proc Natl Acad Sci USA* **91**: 10394–10398

Jennerwein MM, Eastman A and Khokhar AR (1991) The role of DNA repair in resistance of L1210 cells to isomeric 1,2-diaminocyclohexaneplatinum complexes and ultraviolet irradiation. *Mutat Res* **254**: 89–96

Jones JC, Zhen WP, Reed E, Parker RJ, Sancar A and Bohr VA (1991) Gene-specific formation and repair of cisplatin intrastrand adducts and interstrand cross-links in Chinese hamster ovary cells. *J Biol Chem* **266**: 7101–7107

Karran P and Bignami M (1994) DNA damage tolerance, mismatch repair and genome instability. *Bioessays* **16**: 833–839

Kat A, Thilly WG, Fang WH, Longley MJ, Li GM and Modrich P (1993) An alkylation-tolerant, mutator human cell line is deficient in strand-specific mismatch repair. *Proc Natl Acad Sci USA* **90**: 6424–6428

Koi M, Umar A, Chauhan DP, Cherian SP, Carethers JM, Kunkel TA and Boland CR (1994) Human chromosome 3 corrects mismatch repair deficiency and microsatellite instability and reduces *N*-methyl-*N*′-nitro-*N*-nitrosoguanidine tolerance in colon tumor cells with homozygous hMLH1 mutation [published erratum appears in *Cancer Res* 1995 **55**: 201]. *Cancer Res* **54**: 4308–4312

Kolodner RD (1995) Mismatch repair: mechanisms and relationship to cancer susceptibility. *Trends Biochem Sci* **20**: 397–401

May A, Nairn RS, Okumoto DS, Wassermann K, Stevnsner T, Jones JC and Bohr VA (1993) Repair of individual DNA strands in the hamster dihydrofolate reductase gene after treatment with ultraviolet light, alkylating agents, and cisplatin. *J Biol Chem* **268**: 1650–1657

Mello JA, Lippard SJ and Essigmann JM (1995) DNA adducts of cis-diamminedichloroplatinum(II) and its trans isomer inhibit RNA polymerase II differentially in vivo. *Biochemistry* **34**: 14783–14791

Mello JA, Acharya S, Fishel R and Essigmann JM (1996) The mismatch-repair protein hMSH2 binds selectively to DNA adducts of the anticancer drug cisplatin. *Chem Biol* **3**: 579–589

Mu D, Tursun M, Duckett DR, Drummond JT, Modrich P and Sancar A (1997) Recognition and repair of compound DNA lesions (base damage and mismatch) by human mismatch repair and excision repair systems. *Mol Cell Biol* **17**: 760–769

Parker RJ, Eastman A, Bostick-Bruton F and Reed E (1991) Acquired cisplatin resistance in human ovarian cancer cells is associated with enhanced repair of cisplatin-DNA lesions and reduced drug accumulation. *J Clin Invest* **87**: 772–777

Rittner K, Stoeppler H, Pawlita M and Sczakiel G (1991) Versatile eucaryotic vectors for strong and constitutive transient and stable gene expression. *Methods Mol Cell Biol* **2**: 176–181

Sheibani N, Jennerwein MM and Eastman A (1989) DNA repair in cells sensitive and resistant to cis-diamminedichloroplatinum(II): host cell reactivation of damaged plasmid DNA. *Biochemistry* **28**: 3120–3124

Umar A, Koi M, Risinger JI, Glaab W, Tindall KR, Kolodner RD, Boland CR, Barrett JC and Kunkel TA (1997) Correction of hypermutability, N-methyl-N′-nitro-N-nitrosoguanidine-resistance and defective DNA mismatch repair by introducing chromosome 2 into human tumor cells with mutations in *MSH2* and *MSH6*. *Cancer Res* **57**: 3949–3955

Vaisman A, Varchenko M and Chaney SG (1997) Correlation between mismatch repair defects and increased replicative bypass in cisplatin resistant cell lines. *Proc Am Assoc Cancer Res* **38**: 312

Vaisman A, Varchenko M, Umar A, Kunkel TA, Risinger JI, Barrett JC and Chaney SG (1998) Defects in hMSH6, but not hMSH3, correlate with increased resistance and enhanced replicative bypass of cisplatin, but not oxaliplatin, adducts. *Proc Am Assoc Cancer Res* **39**: 159

Zamble DB and Lippard SJ (1995) Cisplatin and DNA repair in cancer chemotherapy. *Trends Biochem Sci* **20**: 435–439

*© Cancer Research Campaign 1999*
